# Rate of HIV transmission and associated factors among HIV-exposed infants in selected health facilities of East and West Gojjam Zones, Northwest Ethiopia; retrospective cohort study

**DOI:** 10.1186/s12879-017-2578-3

**Published:** 2017-07-06

**Authors:** Nurilign Abebe Moges, Getachew Mullu Kassa, Dube Jara Boneya

**Affiliations:** College of Health Sciences, Debre Markos University, P.O.BOX: 269, Debre Markos, Ethiopia

**Keywords:** PMTCT, MTCT, HIV, Exposed infant, Ethiopia

## Abstract

**Background:**

In 2014, there were 170,000 new HIV-infected children globally. The rate of HIV transmission from mother to child in Ethiopia was 18%. Though there are a number of HIV-related studies conducted in Ethiopia, there is a scarcity of evidence on the rate of mother to child transmission. So, the aim of this study was to determine the rate of HIV transmission and associated factors among HIV-exposed infants in selected health facilities in East and West Gojjam Zones, Northwest Ethiopia.

**Methods:**

Retrospective cohort study design was conducted. A total of 305 exposed infant- and mother pairs were included in this study. Data were collected from seven selected health facilities in East and West Gojjam Zone, Northwest Ethiopia. The study included a four-year duration PMTCT data, registered from July/2011 to July/2015. Data was collected using a prepared checklist. Data was entered using EpiData and analyzed using SPSS software. Descriptive, bivariate and multiple variable logistic regression analysis were conducted. A *p*-value less than 0.05 were used to declare statistical significant association.

**Result:**

Three hundred five infants and their mothers were included in this study. The mean age of mothers was 27.4 with a standard deviation of 4.3 years. The majority, 96.4% of infants were on exclusive breastfeeding before six months. The rate of HIV transmission at the end of 24 months were 5.9% (95% CI: 3.9%–7.9%). The number of positive children was reduced from 14 (10.29%) to 4(2.37%) due to the program shift from option A to option B+. Factors which were associated with transmission of HIV from mother to child were; children who were born from older mothers (AOR = 5.4, 95% CI = 1.15, 25.70), and infants whose mother couldn’t get PMTCT intervention (AOR = 15.95, 95% CI = 3.35, 75), and mothers who became pregnant after they knew they were HIV positive (AOR = 0.22, 95%CI = 0.049,096).

**Conclusions:**

There is significant progress on the reduction of the rate of HIV transmission from mother to child in Ethiopia. Age of the mother, status of the mother at an entry to PMTCT program and presence of PMTCT interventions were significant factors associated with HIV transmission. Hence, the above factors should be given due emphasis on controlling HIV transmission from mother to child.

## Background

Globally, there was a total of 170,000 new HIV-infected children in 2014. The rate of mother to child transmission (MTCT) was reduced by 48% between 2009 and 2014. This was achieved after 8 out of 10 pregnant women received antiretroviral (ARV) medicine to prevent mother to child HIV transmission [1]. MTCT of HIV accounts for 20% of all HIV transmissions [2].

The average rate of HIV transmission from mother to child was 18% among 21 high priority countries including Ethiopia [1]. The high rate of HIV morbidity and mortality among pregnant, lactating women and their infants is still the main health challenge in Ethiopia [3]. In Ethiopia, there are 367,000 people including 23,400 children who are taking antiretroviral therapy. ART coverage for an adult is 76%, while it is still very low for children (23.5%) [3, 4]. In August 2012, the country adopted WHO PMTCT programmatic shift, option B+ strategy. The program recommends lifelong antiretroviral therapy (ART) for all HIV-positive women who are pregnant and breastfeeding, regardless of a cluster of differentiations 4 (CD4) count or clinical stage [3].

The percentage of women who are accessing PMTCT service in Ethiopia was 25.5% in 2010 and it increased to 73% in 2015 [3]. The post-2015 HIV priorities are of high impact interventions that dramatically reduce the annual new infection and save many lives which also pave the path to ending AIDS in Ethiopia. For this vision, the virtual elimination of mother to child transmission is one of the strategic objectives [5].

The magnitude of children ART coverage is only 12% in 2013, although there were 160,000 HIV positive children in 2013 under age of 15 years old [6]. The recent national study indicated that there was a large population of children living with HIV in Ethiopia [6]. The magnitude of HIV-exposed infants who accessed Nevirapine (NVP) prophylaxis was 34% in 2015 [3].

Antenatal Care based Sentinel HIV Surveillance in Ethiopia in 2014 indicated a 2.2% prevalence of HIV among pregnant women. The prevalence was high among urban resident pregnant women (3.9%) than rural residents (1.14%) [4].

Ethiopia has a high rate of MTCT of HIV transmission [7]. The mother to child transmission rate after breastfeeding was 24% at the end of 2012 among all estimated HIV-positive pregnant women. The rate had increased to more than 30% in the recent years [6]. After the implementation of PMTCT Option B+ program, the rate of HIV transmission from mother to child ranges from 7% to 18% [1, 8, 9]. Other studies conducted in Ethiopia have also shown that the magnitude of HIV-positive children in Ethiopia is still high [8–15].

So, this study was conducted to determine the rate of HIV transmission and associated factors among HIV-exposed infants in selected health facilities in East and West Gojjam Zones, Northwest Ethiopia.

## Methods

### Study design, area and period

The retrospective cohort study design was conducted. The study was conducted among randomly selected health facilities in East and West Gojjam Zones, Amhara region, Northwest Ethiopia. The health facility’s PMTCT registrations showed that there were 471 registered mothers in Debre Markos referral Hospital, more than 375 in Finote Selam Hospital, and 350 in other health centers. The study included a four-year duration PMTCT data, registered from July/2011 to July/2015.

### Eligibility criteria

All HIV-exposed infants and their mothers since the start of PMTCT service in each of the selected health facilities were included in the study. Incomplete cards of exposed infants, mothers, transfer out, lost and those who stopped treatment were excluded from the study.

### Sampling technique and variables of the study

**Fig. 1:**
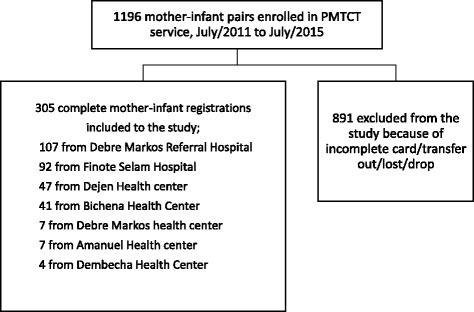
Schematic presentation of the sampling procedure

All mother-infant pairs enrolled in PMTCT service registered from July/2011 to July/2015 in selected health facilities in East and West Gojjam zone were included. The health facilities were randomly selected from all health facilities providing PMTCT services in East and West Gojjam Zones. The selected facilities include; Debre Markos Referral Hospital, Debre Markos Health Center, Amanuel Health Center, Dejen Health Center and Bichena Health Center from East Gojjam zone and Dembecha Health Center and Finote Selam Hospital from West Gojjam zone. A total of 305 complete mother- infant pair PMTCT registrations were included in this study (Fig. 1).

The dependent variable of the study was HIV serostatus of the baby at the end of the follow-up period, 24 months, (positive/negative). The independent variables include; breastfeeding practice, socio-demographic characteristics, mode of delivery, the clinical characteristics of the mother and the characteristics of the infant.

### Operational definition

- ➢ **Sero-status:** if the DBS result indicated positive or negative for HIV during the follow-up period of 24 months as indicated by the infant registration card.
- ➢ **Exposed infant: an** infant who was born from the HIV-positive mother or HIV antibody test positive before 18 months of age [16]**.**
- ➢ **OPTION B+ strategy:** A new WHO program to prevent mother to child transmission of HIV. All HIV-positive pregnant and breastfeeding women are given lifelong antiretroviral therapy, irrespective of the CD4 count and clinical stage of the disease. This is done for their own health, for the prevention of vertical HIV transmission and for additional HIV prevention benefits [1].

### Method of data collection

Structured data capturing checklist was developed from HIV-exposed follow-up card, ANC follow-up registration, adult HIV care and treatment follow-up registration and intake form of Ethiopian national HIV care and treatment package [16]. Data was collected from infant and mother registration cards. First, data was collected from infant’s registration cards and then data was collected from the infant’s mother cards.

### Data quality, processing, and analysis

The standard checklist was used for registration of the infant and mother information. A two-day training was given to the data collectors and supervisors prior to data collection period. Completeness, accuracy, and consistency of the collected data were checked on a daily basis during data collection. Data was entered using EpiData version 3.5 software then exported to SPSS version 20 software for further analysis. Bivariate and multivariable logistic regression analysis was used to identify factors associated with HIV transmission from the mother to the child. A 95% confidence interval of odds ratio and a *p*-value less than 0.05 were used to determine the statistical significance of the independent with the dependent variables.

## Results

### Socio-demographic characteristics

This study included 305 HIV-exposed infants and their mothers enrolled in PMTCT care in selected public health facilities in Northwest Ethiopia. The study included a four-year duration PMTCT data, registered from July/2011 to July/2015. The data was collected from seven health facilities. Most, 107 (35.1%) of the data was collected from Debre Markos referral hospital. More than two third, 205 (67.2%) of mothers were in age group of 25–34 years, with the mean and standard deviation of 27.4 and 4.3 years respectively. One hundred ninety-four (63.6%) of women were married, and 145 (47.5%) were unable to read and write. Almost half, 144 (47.2%) of mothers were not employed. Two hundred ninety (95.1%) of study participants were orthodox religion followers (Table 1).

**Table 1:** Study participant distribution by health facilities for rate of HIV transmission among exposed children in selected facilities of East and West Gojjam Zones, Northwest Ethiopia, 2016

Characteristics	Response	Frequency	Percent
Health facility	Debre Markos Referral Hospital	107	35.1
	Finote Selam Hospital	92	30.2
	Dejen Health Center	47	15.4
	Bichena Health Center	41	13.4
	Debre Markos health center	7	2.3
	Amanuel Health center	7	2.3
	Dembecha Health Center	4	1.3
Age	15–24 years	81	26.6
	25–34 years	205	67.2
	>=35 years	19	6.2
Educational status	Not educated	145	47.5
	Primary	74	24.3
	Secondary	69	22.6
	Tertiary	17	5.6
Occupational status	Employed	161	52.8
	Not employed	47.2	47.2
Disclosure status of the mother	Disclosed	269	88.2
	Not disclosed	39	11.8
For whom disclosed	Husband	175	57.4
	Siblings	59	19.3
	Relatives	82	26.9
	Friends	17	5.6

### Clinical characteristics of the mother

Data was collected on maternal clinical condition during admission and follow-up. Of all mothers at admission, 271 (88.9%), 31 (10.2%) and 3 (0.98%) baseline functional status was working, ambulatory and bedridden respectively. More than half, 159 (52.1%) had one or more types of opportunistic infections during admission. The treatment regimen of the majority, 199 (65.2%) of participants was TDF-3TC-EFV. Half, 155 (50.8%) of participants were at WHO stage 1 and 293 (96.1%) had good adherence to ART medications (Table 2).

**Table 2:** Clinical characteristics of HIV positive mothers in selected health facilities of East and West Gojjam zone, Northwest Ethiopia, 2016

Characteristics	Response	Frequency	Percentage
Opportunistic infection at baseline	Yes	159	52.1
	No	146	47.9
Types of opportunistic disease (more than one disease were observed)	Herpes zoster	44	14.4
	Tuberculosis	23	7.5
	Persistent fever >1 month	15	9.4
	Chronic diarrheal disease	18	11.3
	Weight loss >10%	13	8.2
	Weight loss <10%	22	7.2
	Oral candidiasis	22	7.2
	Bacterial pneumonia	10	3.3
	Toxoplasmosis	6	2
	Cryptococcusis	2	0.7
Baseline WHO stage	stage 1	155	50.8
	stage 2	62	20.3
	stage 3	79	25.9
	stage 4	9	3
Baseline functional status	Working	271	88.9
	Ambulatory	31	10.2
	Bed ridden	3	1
Baseline CD4 count (cell/dl)	Less than 200	96	31.5
	200–350	90	29.5
	351–500	62	20.3
	>500	57	18.7
Treatment regiment	1e (TDF-3TC-EFV)	199	65.2
	Other Regimen^a^	106	34.8
Change in treatment regimen	Yes	58	19
	No	247	81
ART adherence	Good	293	96.1
	Poor	12	3.9

^a^1c (AZT-3TC-NVP), 1d(AZT-3TC-EFV), 1f(TDF-FTC-NVP)

### Obstetrics characteristics of the mother

**Fig. 2:**
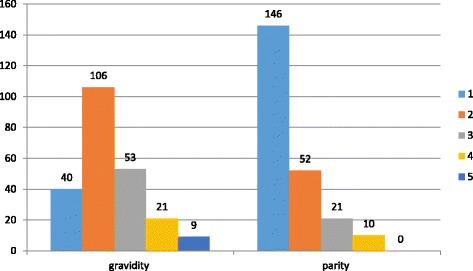
Number of gravidity and parity among HIV-positive mothers in selected health facilities of East and West Gojjam zones, Northwest Ethiopia, 2016

Obstetric history of mothers was also collected. Accordingly, 106 (46.3%) were gravid two for the prospective infants and 146 (47.9%) were parity one (Fig. 2). Only half, 156 (51.1%) of the study participants blood hematocrit were measured. The mean hematocrit value was 38.47% (SD ± 5.6) at first ANC booking and 38.33% (SD ± 4.2) at the fourth ANC visit. One hundred sixty-two (95.3%) mothers were RH positive.

The blood group of mothers were A, B, AB and O for 72 (42.4%), 40 (23.5%), 9 (5.3%), and 49 (28.8%) of mothers respectively. The majority, 220 (97.3%) of mothers have received at least one dose of Tetanus toxoid (TT) vaccination and 111 (49.1%), 49 (21.7%), 34 (15%) and 32 (14.2%) of mothers had fourth, third, second and first ANC follow-up respectively. More than two third of pregnant women, 159 (75.7%) took iron-folate supplementation during the antenatal visit. The common mode of delivery was spontaneous vaginal delivery, 106 (87.6%), while vacuum assisted delivery account 8 (6.6%), caesarean section 5 (4.1%) and 2 were delivered by assisted delivery with forceps. Most, 115 (95.8%) of infants were delivered at term (gestational age 37 weeks to 42 weeks), while 5 (4.2%) were preterm babies. Only 25 (8.2%) had received vitamin A.

### Behavioral characteristic of respondents

Mothers’ risk sexual behavior and overall HIV-related knowledge were measured. The finding of this study showed that 198 (64.9%) of mothers had risk sexual behavior. Mothers who had good knowledge of HIV disease were 89 (29.2%) and the rest 216 (70.8%) had poor knowledge. In line with this, 195 (63.9%) mothers had poor knowledge regarding ways of HIV transmission and 242 (79.3%) had poor knowledge about prophylaxis to opportunistic infections. All women had good knowledge on the importance of ART medication adherence.

### Rate of HIV transmission and characteristics of exposed infants

At the end of 18 months and above of infant follow-up, 18 (5.9%) of HIV-exposed infants were confirmed to be HIV-positive (95% CI: 3.88%–7.9%). More than half, 173 (56.7%) children were male. The mean birth weight was 3.01 kg while average weight at enrolment was 5.18 kg. Growth pattern of 303 (99.3%) infants was normal. Most, 256 (83.9%) of exposed infants got at least one type of immunization. The majority, 294 (96.4%) of infants were on exclusive breastfeeding until 6 months of their life (Table 3).

**Table 3:** Characteristics of HIV-exposed infants and their mother on follow-up in selected health facilities of East and West Gojjam zone, Northwest Ethiopia, 2016

Characteristics	Response	Frequency	Percentage
Sex of child	Male	173	56.7
	Female	132	43.3
Age of enrolment for care	Less than or equal to 6 weeks	211	69.2
	Greater than 6 weeks	94	30.8
Place of birth	Home	39	12.8
	Health institution	266	87.2
Where born from health institutions	Debre Markos referral Hospital	88	28.9
	Finote Selam Hospital	62	20.3
	Other health institutions^a^	116	38
First DBS test result	Positive	19	6.2
	Negative	286	93.8
Time first DBS taken after birth	Less than or equal to 6 weeks	221	72.5
	Greater than 6 weeks	84	27.5
Infant prophylaxis	Given	281	92.1
	Not given	24	7.9
Type of prophylaxis given to the infant	Daily NVP	192	68
	NVP + AZT	110	32
Father HIV status	Positive	205	67.2
	Negative	36	11.8
	Unknown	64	21
Father status	Alive	277	90.8
	Dead	28	9.2
Immunization status of the infant for routine EPI	Immunized	256	83.9
	Not immunized	49	16.1
Entry to PMTCT	During ANC	70	23
	Postpartum	38	12.5
	Known positive	197	64.6
PMTCT intervention	None	24	7.9
	HAART	281	92.1
Birth weight (*n* = 157)	2.5–4.0 kg	129	82.2
	less than 2.5 kg	28	17.8
Infant feeding practice <6 months	Exclusive breast feeding	294	96.4
	Exclusive replacement feeding	9	3
	Mixed feeding	2	0.7
Infant breastfeeding practice after 6 month	Breastfeeding and complementary feeding	250	82
	Replacement feeding	55	18

^a^Debre Markos health, Bichena, Dejen, Dembecha and Amanuel health centres

### Factors associated with rate of HIV transmission

Socio-demographic characteristics of the mother, father, and infants were assessed with the possible association of HIV infection among exposed infants. The bivariate analysis showed that the infants who were born at the referral hospital, maternal age greater than 27.4 years were associated with HIV positive outcome of infants (Table 4).

**Table 4:** Bivariate logistic regression analysis of socio-demographic factors with rate of HIV transmission to exposed infants among selected health facilities of East and West Gojjam zone, Northwest Ethiopia, 2016

Variable		Frequency	COR at 95% CI	*p*-value
Maternal age (mean = 27.4, SD = ±4.3)	Less or equal to 27.4 years	155	1	
	Greater than 27.4 years	150	5.6 (1.6–19.9)	0.007
Marital status	Married	194	1	
	Single, divorced, widowed, separated	111	1.4 (0.55–3.7)	0.47
Educational status	Not educated	145	1	
	Primary education	74	0.88 (0.3–2.6)	0.82
	Secondary education	69	0.18 (0.2–1.4)	0.1
	Tertiary education	17	0.76 (0.9–6)	0.8
Occupational status of the mother	Employed	161	1	
	Not employed	144	0.89 (0.34–2.31)	0.81
Disclosure status	Yes	255	1	
	No	32	2.28 (071–7.34)	0.68
Father HIV status	Positive	205	1	
	Negative	36	1.15 (0.24–5.47)	0.86
	Unknown	64	2 (0.7–5.79)	0.19
Father status	Alive	277	1	
	Dead	28	1.26 (0.27–5.76)	0.77
Sex of child	Male	173	1	
	Female	132	0.36 (0.11–1.10)	0.07
Place of birth	Home	39	1	
	Debre Markos referral hospital	88	0.16 (0.3–0.86)	0.032
	Finote selam Hospital	62	0.47 (0.12–1.87)	0.28
	Other health centers^a^	116	0.44 (0.13–1.47)	0.18

^a^Debre Markos health center, Bichena, Dejen, Dembecha and Amanuel health centers

From the clinical characteristics; poor maternal ART adherence, mothers who were known HIV positive before pregnancy, mothers who were not on ART and mothers who use cotrimoxazole preventive therapy (CPT) were the identified factors (Table 5).

**Table 5:** Bivariate logistic regression analysis of maternal clinical factors with rate of HIV transmission for exposed infants among selected health facilities of East and West Gojjam zone, Northwest Ethiopia, 2016

Variables	Response	Frequency	COR at 95% CI	*P*-value
Functional status of the mother at baseline	Working	271	1	
	Ambulatory/ Bedridden	34	0.45 (0.06–3.51)	0.45
Opportunistic infection at baseline	Yes	159	1	
	No	146	1.4 (0.53–3.62)	0.50
Maternal CD4 count (cell/dl)	<=350	186	1	
	>350	119	0.58 (0.2–1.7)	0.32
ART adherence of the mother	Good	293	1	
	Poor	12	9.96 (2.68–37.10)	0.001
Baseline WHO HIV stage	Stage I and II	217	1	
	Stage III and IV	88	1.25 (0.45–3.44)	0.67
Recent ART regimen	TDF-3TC-EFV	199	1	
	Others^a^	103	1.25 (0.47–3.3)	0.66
Iron/folic acid	Given	155	1	
	Not given	47	3.3 (0.79–13.70)	0.10
Place of birth	Health facility	266	1	
	Home	39	2.86 (0.96–8.53)	0.059
Risk sexual behavior	Yes	66	1	
	No	182	0.17 (0.02–1.33)	0.09
Entry to PMTCT	ANC	193	1	
	Post-partum	64	2.84 (0.91–8.9)	0.07
	Known positive	30	0.22 (0.60–0.81)	0.022
PMTCT intervention	HAART	281	1	
	None	24	24.38 (8.3–71.3)	0.000
Maternal CPT	Yes	298	1	
	No	7	14.2 (2.90–69)	0.001
Mother on ART during labor	Yes	265	1	
	No	40	8.26 (3.05–22.36)	0.000

^a^1c (AZT-3TC-NVP), 1d(AZT-3TC-EFV), 1f(TDF-FTC-NVP)

Infants who were enrolled for treatment and care later than 6 weeks, ARV prophylaxis, immunization status and the initiation of option B+ PMTCT program were associated with HIV positivity on bivariate analysis (Table 6).

**Table 6:** Bivariate logistic regression analysis of infant clinical factors for exposed infants among selected health facilities of East and West Gojjam zone, Northwest Ethiopia, 2016

Variables	Response	Frequency	COR at 95% CI	*P*-value
Age at enrolment	At 6 weeks	211	1	
	>6 weeks	94	3.86 (1.45–10.31)	0.007
Birth weight	Low birth weight	28	1.56 (0.16–15.5)	0.71
	Normal weight	129	1	
ARV prophylaxis’s	Given	282	1	
	Not given	23	10.78 (3.68–31.53)	0.000
Immunization status for routine EPI in Ethiopia	Vaccinated	256	1	
	Not vaccinated	49	3.71 (1.36–10.12)	0.01
Enrolled in to care	Before Option B+ strategy	136	1	
	After option B+ strategy	169	0.21 (0.07–0.6)	0.007
Infant feeding practice <6 months	Exclusive breastfeeding	294	1	
	Exclusive replacement feeding	11	3.86 (0.77–19.34)	0.10
Infant breastfeeding practice after 6 month	Breastfeeding +complementary feeding	250	1	
	Exclusive replacement feeding	55	0.9 (0.25–3.24)	0.88

Variables from bivariate logistic regression with a *p*-value less than 0.25 were fitted into the multivariable logistic regression model. These variables were maternal age, place of birth, sex of the child, entry status to PMTCT, the presence of PMTCT intervention, mother on HAART, a mother who uses CPT, the age of enrolment to care, ARV prophylaxis, vaccination status, availability of PMTCT program and breastfeeding practice.

The multivariable logistic regression analysis showed that; children who were born from older mothers (AOR = 5.4, 95% CI = 1.15–25.70), infants whose mothers didn’t get PMTCT intervention (AOR = 15.95, 95% CI = 3.35–75), and mothers who become pregnant after they were aware of their HIV status (AOR = 0.22, 95% CI = 0.049–096) were the factors associated HIV transmission to the infant (Table 7).

**Table 7:** Multivariate logistic regression analysis factors associated with rate of transmission of HIV-exposed infant among selected health facilities of East and West Gojjam Zones, Northwest Ethiopia, 2016

Variables	Response	Frequency	COR (95% CI)	AOR (95% CI)	*P*-value
Maternal age (mean = 27.4, SD = +/−4.3)	Less or equal to 27.4 years	155	1	1	
	Greater than 27.4 years	150	5.6 (1.6–19.9)	5.4 (1.15–25.70)	0.033
Place of birth	Home	39	1	1	
	Debre Markos referral hospital	88	0.16 (0.3–0.86)	0.22 (0.02–2.23)	0.19
	Finote selam hospital	62	0.47 (0.12–1.87)	1.14 (0.15–9.03)	0.90
	Other health centers	116	0.44 (0.13–1.47)	0.71 (0.14–3.63)	0.68
ART adherence of the mother	Good	293	1	1	
	Poor	12	9.96 (2.68–37.10)	5.76 (0.75–44.4)	0.84
Entry to PMTCT intervention	During ANC/ during pregnancy	193	1	1	
	During post-partum period	64	2.84 (0.91–8.9)	0.16 (0.02–1.72)	0.13
	Known positive/ prior to pregnancy	30	0.22 (0.60–0.81)	0.22(0.049–096)	0.045
PMTCT intervention	HAART	281	1	1	
	None	24	24.38(8.3–71.3)	15.95 (3.35–75)	0.001
Maternal Cotrimoxazole prophylaxis therapy	Yes	298	1	1	
	No	7	14.2 (2.90–69)	2.69 (0.23–32.18)	0.43
Mother was on ART during labor	Yes	265	1	1	
	No	40	8.26 (3.05–22.36)	6.37(0.85–47.97)	
Enrolled in to PMTCT care	Before Option B+ strategy	136	1	1	
	After Option B+ strategy	169	0.21 (0.07–0.6)	0.22 (0.04–1.07)	0.055
ARV prophylaxis was given for the child	Given	282	1	1	
	Not given	23	10.78 (3.68–31.53)	2.48(0.50–12.32)	0.26
Immunization for the baby/of any type	Vaccinated	256	1	1	
	Not vaccinated	49	3.71 (1.36–10.12)	0.86 (0.14–5.46)	0.87
Type of breastfeeding <6 month	Exclusive breastfeeding	294	1	1	
	Exclusive replacement feeding	11	3.86 (0.77–19.34	4.48(0.39–51.73)	0.21

## Discussion

This study was conducted to determine the rate of HIV transmission and associated factors among HIV-exposed infants in selected health facilities in East and West Gojjam Zone, Northwest Ethiopia.

The rate of HIV transmission from mother to child was found to be 5.9% at the end of the 24 months follow-up. This finding is higher than a study done in South Africa, Ukraine, Haiti and Congo [17–20]. However, it is lower than a study done in South Gondar, Northwest Ethiopia (10.1%), Southwest Ethiopia (9.6%) and Gondar University Hospital [8, 9, 13]. The difference might be due to the time difference between the studies, in which this study was conducted at a time of high ART coverage for pregnant and lactating women than the previous studies in Ethiopia. The difference in the sociodemographic characteristics could also contribute to the variation in findings between the studies.

This study also showed a reduced rate of mother to child transmission of HIV from 10.29%, prior to the introduction of the option B+ strategy, to 2.37% (*p* < 0.007) after its introduction. This could be because of PMTCT strategy, government policies, system strengthening and other interventions that might have contributed to the reduction of the MTCT of HIV. This finding is supported by a study conducted in Zambia and Malawi [21, 22]. This shows that the strategy is good foot step for elimination plan of vertical HIV transmission below 2% in Ethiopia [23].

Infants born from maternal age above 27.4 years were 5.4 times more likely to be HIV-positive at the end of 24 months compared to mothers who were relatively younger, less than 27.4-year-old. This might be because older mothers in the study area were less educated than younger women, which could have a negative effect on health seeking behavior of mothers. This finding is similar to study in northwest Ethiopia in which infants born from age of above 25 years were more likely to have DBS test result positive [8]. A study conducted in Kinshasa-DRC and Malawi also revealed that younger age mothers had a better infant outcome than older mothers [22, 24]. However, there was no significant association between clinical adherences for prevention of HIV from mother to child with caregivers age in Cameroon [25].

A mother who became pregnant after they knew their HIV serostatus were 0.22 times less likely to have HIV positive child compared to those who knew their HIV status during antenatal care or post-partum, after pregnancy. A similar study in Ethiopia reported a higher risk of infection due to delayed HIV diagnosis of the mother [8]. Studies conducted in Ukraine and University of Gondar Hospital, Ethiopia also showed similar finding [13, 17]. The possible explanation for a higher rate of MTCT of HIV in mothers who are newly diagnosed compared with known HIV-positive mothers could be; known the positive mother has good clinical adherence so they can benefit from antiretroviral drugs. HIV positive mother on the antiretroviral drug will be with the low viral load so that there is less probability of transmitting to the child through pregnancy, childbirth, and breastfeeding. Mother on chronic HIV care would be with better knowledge of PMTCT and adhered to the recommended prevention mechanism [26].

Mothers with no PMTCT intervention were more likely to have HIV positive children at the end of follow-up. This result is similar to study conducted in other parts of Ethiopia, Cameroon, Tanzania, and Malawi. These studies showed a higher risk of HIV transmission from ANC and post-partum diagnosis compared to known positive mothers [8, 13, 22, 27, 28]. This can be explained in line with the advantage of antiretroviral drug.

The current study has certain limitations. Since it was a retrospective cohort, some of the registrations were not complete. Due to this, some variables which may affect the MTCT of HIV were not documented in the registration forms and were excluded from the analysis. The identified factors in this study are associational only and it may be difficult to make a causal inference.

## Conclusions

The rate of HIV positivity of exposed infants is decreasing meaningfully in the study area. A good progress for virtual elimination of mother to child transmission of HIV is observed. HIV-positive women who become pregnant after they knew their HIV serostatus and who were on clinical care before pregnancy had the highest probability of HIV-negative child. Older age HIV positive mothers were more likely to have HIV positive child than younger mothers. A mother with no PMTCT intervention has a high risk of having HIV-positive baby.

The high rate of risky sexual behavior and poor knowledge of HIV transmission among study participants was also observed. This needs further study to identify possible reasons for high rate of risky sexual behavior among HIV-positive women. Program planners and policy makers at a regional and national level should give more emphasis to the factors identified in this study. Clinicians should give due attention to older mothers during clinical follow-up, and proper documentation of care provided to HIV-positive women and their infants should be encouraged. A nationwide representative study on determinants of MTCT of HIV in resource-limited settings is also recommended.

## Abbreviations

ART: Antiretroviral therapy
CPT: Cotrimoxazole preventive therapy
DBS: Dried blood sample
HIV: Human immune deficiency virus
MTCT: Mother to child transmission
PMTCT: Prevention of mother to child transmission
WHO: World Health Organization
